# Achievement of European Society of Cardiology/European Atherosclerosis Society lipid targets in very high-risk patients: Influence of depression and sex

**DOI:** 10.1371/journal.pone.0264529

**Published:** 2022-02-25

**Authors:** Elizabeth A. Ellins, Daniel E. Harris, Arron Lacey, Ashley Akbari, Fatemeh Torabi, Dave Smith, Geraint Jenkins, Daniel Obaid, Alex Chase, Ann John, Michael B. Gravenor, Julian P. Halcox

**Affiliations:** 1 Swansea University Medical School, Singleton, Swansea, United Kingdom; 2 Swansea Bay University Health Board, Port Talbot, United Kingdom; 3 Population Data Science, Health Data Research UK, Swansea University Medical School, Swansea, United Kingdom; 4 Public Health Wales NHS Trust, Cardiff, United Kingdom; University Hospital Zurich: UniversitatsSpital Zurich, SWITZERLAND

## Abstract

**Aims:**

To explore differences in the use of lipid lowering therapy and/or achievement of lipid guideline targets in patients with and without prior depression and influence of sex in very high-risk coronary patients.

**Methods & findings:**

A retrospective observational cohort study was conducted using individual-level linked electronic health record data in patients who underwent percutaneous coronary intervention (2012–2017) in Wales. The cohort comprised of 13,781 patients (27.4% female), with 26.1% having prior depression. Lipid levels were recorded in 10,050 patients of whom 25% had depression. History of depression was independently associated with not having lipids checked (OR 0.79 95%CI 0.72–0.87 p<0.001). Patients with prior depression were less likely to achieve targets for low density lipoprotein cholesterol (LDL-C <1.8mmol/l), non-high density lipoprotein cholesterol (non-HDL-C <2.6mmol/l) and triglycerides (<2.3mmol/l) than patients without depression (OR 0.86 95%CI 0.78–0.96 p = 0.007, OR 0.80 95%CI 0.69–0.92 p = 0.003 & OR 0.69 95CI% 0.61–0.79 p<0.001 respectively). Females were less likely to achieve targets for LDL-C and non-HDL-C than males (OR 0.55 95%CI 0.50–0.61 p<0.001 & OR 0.63 95%CI 0.55–0.73 p<0.001). There was an additive effect of depression and sex; females with depression were not only least likely to be tested (OR 0.74 95%CI 0.65–0.84 p<0.001) but also (where levels were known) less likely to achieve LDL-C (OR 0.47 95%CI 0.41–0.55 p<0.001) and non-HDL-C targets (OR 0.50 95%CI 0.41–0.60 p<0.001). It was not possible to look at the influence of medication adherence on achievement of lipid targets due to limitations of the use of anonymised routinely-held clinical care data.

**Conclusion:**

Patients with prior depression were less likely to have their lipids monitored and achieve guideline targets within 1-year. Females with depression are the least likely to be tested and achieve lipid targets, suggesting not only a greater risk of future events, but also an opportunity to improve care.

## Introduction

Common mental disorders such as depression and anxiety, are increasingly recognised to play a role in our physical health. The association between depression and increased risk of developing cardiovascular disease (CVD), and recurring coronary events is well known, but not always considered when managing these patients [1, 2].

Percutaneous coronary intervention (PCI) is an effective revascularisation treatment for patients with acute coronary syndrome and stable coronary artery disease. However, patients with depression have been shown to have worse outcomes following PCI [3]. The reasons for this are not fully understood. However, differences in effectiveness of management of conventional risk factors may be a contributing factor, as in patients with severe mental illness [4, 5]. Whilst some evidence suggests that those with depression may also be less likely to adhere to medication and reach targets for lipids and blood pressure [6].

Another important consideration is sex. Females are known to have worse outcomes than males following PCI [7]. They are also less likely to have their CV risk factors treated as intensively as males and post PCI to meet lipid guidelines targets, as well as more likely to have depression [8, 9]. However, the combined impact of both sex and depression on risk factor management in very high-risk patients is not fully understood.

We have previously shown in a cohort of PCI patients that only 48% and 23% met the European Society of Cardiology/ European Atherosclerosis Society (ESC/EAS) 2016 and 2019 lipid guidelines targets for low density lipoprotein cholesterol (LDL-C) [8]. Importantly, it is recognised that women are both more likely to experience depressive illness and less likely to receive optimal treatment for CVD than men, the incremental impact of sex and depression on risk factor management in cardiac patients has not been fully examined at population scale. Therefore, in this study, we aimed to investigate the use of lipid lowering therapy and/or achievement of guideline targets for lipid levels in patients with prior depression and the incremental influence of sex on these relationships in patients with coronary artery disease, after undergoing PCI.

## Methods

We carried out a retrospective observational cohort study using individual-level linked anonymised electronic health record (EHR) data for patients undergoing percutaneous coronary intervention between January 2012 and December 2017. Access to data and linkage was through the privacy-protecting trusted research environment (TRE) the Secure Anonymised Information Linkage (SAIL) Databank [10, 11]. PCI was identified from the population-scale national secondary care data (Patient Episode Database for Wales [PEDW]) and the date of the first PCI during the study period for each patient was assigned as the index date. We included a follow-up period of 1year from date of discharge from the index admission (time zero). Presence of hypertension, ischaemic heart disease, chronic kidney disease (CKD) stage 4+, depression, anxiety, severe mental illness, prescriptions for lipid lowering and antidepressant therapy and recorded lipid levels were identified from primary care data from the Welsh Longitudinal General Practice (WLGP) data. Both primary and secondary care data sources were used to describe prior history of myocardial infarction and prior or contemporary (at time of index admission) diagnosis of peripheral vascular disease (PVD), heart failure, diabetes mellitus and ischaemic stroke. The secondary care data was also used to identify whether the index admission was for an acute coronary syndrome (ACS) or stable coronary artery disease. The Welsh Demographic Service Dataset (WDSD) contains anonymised area level residential information for residences in Wales from the Lower-layer Super Output Area (LSOA) version 2001, and linkage of the LSOA to the area-based deprivation measure Welsh Index of Multiple Deprivation (WIMD) 2011, an indicator of socioeconomic status. Inclusion criteria for the study included being ≥18 years of age at time of index PCI, with a minimum of 90-days of both pre-PCI and follow-up data available in the WLGP data and free from severe mental illness (SMI) or anxiety pre and post-PCI.

### Access to data and ethical approval

The data used in this study are available in the SAIL Databank at Swansea University, Swansea, UK. All proposals to use SAIL data are subject to review by an independent Information Governance Review Panel (IGRP). Before any data can be accessed, approval must be given by the IGRP. The IGRP gives careful consideration to each project to ensure proper and appropriate use of SAIL data. When access has been approved, it is gained through a privacy-protecting safe haven and remote access system referred to as the SAIL Gateway. SAIL has established an application process to be followed by anyone who would like to access data via SAIL https://www.saildatabank.com/application-process. This project was approved by the IGRP at Swansea University (SAIL project number 0915). Participant consent was not required by the IGRP as all data was anonymised prior to the study.

### Depression characterisation

Primary care records were used to identify patients who had a record of any of the following: a diagnosis of depression or mixed anxiety and depression, anxiety, severe mental illness, depressive symptoms, anxiety symptoms, prescriptions for antidepressants or anxiolytics. A list of diagnostic (Read) codes was created based on previous work from our group [12]. Patients were categorised as having a history of depression prior to their PCI if they met the following criteria (to be identified as depression forthwith), a diagnosis of depression or mixed anxiety and depression in their medical history up until the index date of their first PCI, or a record of depressive symptoms together with a prescription of antidepressants within their medical history. Depressive symptoms were included in the depression categorisation to reflect changes within coding behaviour as specified in John et al. [12], with only those patients with both symptoms and a prescription for antidepressants being included in the depressed group. This approach has been validated through linkage to survey data by John et al. [12] Those patients prescribed antidepressants, but without a record of depression diagnosis or symptoms were not categorised as depressed. Patients with severe mental illness were excluded, as were those who had a diagnosis of anxiety without depression. All mental health diagnostic (Read) codes used for inclusion and exclusion purposes are listed in S1 Table.

### Lipid levels

The time to the first lipid profile and the lowest LDL-C (estimated from the Friedewald formula), non-high-density lipoprotein cholesterol level (non-HDL-C) and triglycerides (TG) between 28- and 365-days following discharge was noted [13]. Prescriptions for lipid lowering therapy (LLT) including statins, ezetimibe, fibrates and prescription grade N-3 supplements were identified. PCSK-9 inhibitors were not included as they were only approved for use within the UK National Health Service in 2016 and their use limited to specialist clinics. LLT prescribed in the 90-days immediately post-discharge and within 90-days prior to the lowest LDL-C, and non-HDL-C readings were classified as high intensity statin (HI-statin; atorvastatin ≥40mg/d and rosuvastatin ≥20mg/d), non-high-intensity (NI-statin; any other statin prescription), a combination of ezetimibe and/or fibrate with either HI- or NI-statin (combination statin), other treatments including ezetimibe and/or fibrate (other treatment) without a co-prescription of a statin, or no treatment. LLT prescribed within the 90-days prior to the lowest TG level were classified as statin (either HI- or NI-statin); combination fibrate and/or N-3 with a statin (combination statin), fibrate and/or N-3 (other), or no treatment. We identified the number (and proportion) of patients achieving the ESC/EAS 2016 and 2019 lipid guideline levels of (i) LDL < or ≥ 1.8 and 1.4 mmol/L; (ii) non-HDL < or ≥ 2.6 and 2.2 mmol/L; (iii) TG < or ≥ 2.3 and 1.5 mmol/L and their respective LLT regimens [14, 15].

### Statistical analysis

Variables are presented as mean (standard deviation [SD]) for continuous and frequency (percentage) for categorical variables. Comparison between those with and without depression and/or lipid profiles were carried out using a two-sample t-test or chi square as appropriate (TG’s were log transformed and the geometric mean reported). The main outcome variables explored were i) having a lipid test post PCI and ii) reaching target lipid levels within one year of PCI. Binary logistic regression was used to explore the relationships between depression, as the primary exposure, the odds of being prescribed a statin prior to PCI and the two main outcome variables. Adjustments were made for age, sex, and relevant clinical variables (Table 1). We also tested for the interaction between sex and depression. The Wald Χ^2^ statistic was used to indicate significance of the coefficients. As a sensitivity analysis of model selection, and due to the potential for inter-dependency of several variables in this data, an estimate of the effect of depression was also obtained using a minimally sufficient set of variables based on assumed directed acyclic graphs (DAGs) (S1 File). The incremental impact of depression and sex on the odds of having a lipid test and achieving lipid targets was explored through logistic regression. Analyses were carried out using SPSS Statistics Software version 26 and R version 3.5.

**Table 1:** Cohort characteristics and comparison between those with and without depression.

	Depression n = 3,594	No depression n = 10,187	p
Percentage of total cohort (%)	26.1	73.9	
Mean age years (SD)	61.5 (11.6)	66.4(11.5)	<0.001
Characteristic n (%)			
Female	1,335 (37.1)	2,445 (24.0)	<0.001
Obese	1,342 (40.3)	3,002 (33.0)	<0.001
Current smoker	1,698 (47.2)	3,140 (30.8)	<0.001
Deprivation index n (%)			
1 (most deprived)	1,097 (30.5)	1,942 (19.1)	<0.001
2	749 (20.8)	1,969 (19.3)	
3	648 (18.0)	2,061 (20.2)	
4	478 (13.3)	1,780 (17.5)	
5 (least deprived)	506 (14.1)	2,106 (20.7)	
Past medical history n (%)			
Hypertension	1,510 (42.0)	4,209 (41.3)	0.47
Ischaemic heart disease	1,051 (29.2)	2,618 (25.7)	<0.001
Myocardial Infarction	667 (18.6)	1,563 (15.3)	<0.001
Coronary revascularisation	413 (11.5)	877 (8.6)	<0.001
Ischaemic stroke	279 (7.8)	632 (6.2)	0.001
Heart Failure	480 (13.4)	1,494 (14.7)	0.054
Peripheral vascular disease	264 (7.3)	593 (5.8)	0.001
Diabetes Mellitus	974 (27.1)	2,249 (22.1)	<0.001
Chronic Kidney Disease Stage 4+	57 (1.6)	110 (1.1)	0.017
Medications 1 year before admission n (%)			
Statins	1,843 (51.3)	4,782 (46.9)	<0.001
Other LLT	149 (4.1)	273 (2.7)	<0.001
SSRI’s	1,369 (38.1)	225 (2.2)	<0.001
Tricyclics	542 (15.1)	627 (6.2)	<0.001
Other antidepressants	525 (14.6)	96 (0.9)	<0.001

Obesity defined as body mass index greater than 30 kg/m^2^ LLT: lipid lower therapy; SSRI’s: selective serotonin reuptake inhibitor.

## Results

### Population characteristics

The study inclusion criteria were met by 13,781 participants (for cohort chart see S1 Fig), of these, 3,594 (26.1%) were categorised as having a record of depression in their medical history prior to PCI. Participants in the depression group were younger, more likely to be female, have a diagnosis of diabetes mellitus and a diagnosis of cardiovascular disease prior to PCI than those without depression (Table 1). Depression was associated with being prescribed statin treatment pre-PCI, after adjustment for covariates (OR 1.21 95% CI 1.10–1.32 p<0.001. S2 Table).

### Testing of lipid levels post percutaneous coronary intervention

Lipid levels were documented in 10,050 (72.9%) participants (characteristics of those tested and not tested shown in S3 Table). Participants with depression were more likely to have no recorded lipids (29 vs 25% p<0.001). Those patients who were older, female, or with chronic kidney disease, peripheral vascular disease, atrial fibrillation, prior ischaemic stroke, or acute coronary syndrome indication for index PCI were less likely to have a lipid level recorded. Patients prescribed LLT post discharge were more likely to undergo lipid testing than those not in receipt of LLT prescription during the 12 months post-PCI. Multivariable logistic regression modelling showed that those with depression were less likely to have their lipid levels checked during the first year of follow-up (OR 0.79 95%CI 0.72–0.87 p<0.001) (Table 2). Very similar results were obtained using DAG model selection, S4 Table).

**Table 2:** Full mutually adjusted binary logistic regression identifying variables associated with having lipid levels documented during the first year of follow up, post-percutaneous coronary intervention.

Covariate	Odds ratio	95% C.I.	p
Age	0.99	0.99–1.00	0.004
Female	0.95	0.87–1.04	0.24
Diabetes	1.70	1.54–1.89	<0.001
Contemporary acute coronary syndrome	1.43	1.30–1.56	0.000
Hypertension	0.96	0.89–1.05	0.37
Chronic kidney disease	0.49	0.35–0.69	<0.001
Heart failure	1.02	0.91–1.15	0.75
Ischaemic stroke	0.88	0.75–1.03	0.11
Peripheral vascular disease	0.71	0.61–0.83	<0.001
Atrial fibrillation	0.87	0.77–1.00	0.046
Lipid lowering therapy (LLT)			<0.001
No LLT	REF		
High statin	3.67	3.14–4.30	
Low statin	4.39	3.75–5.13	
Other	5.03	3.20–7.90	
Statin + other	4.03	2.95–5.51	
Deprivation index			0.042
1 (most deprived)	0.86	0.76–0.98	
2	0.89	0.79–1.01	
3	0.97	0.86–1.10	
4	1.02	0.89–1.16	
5 (least deprived)	REF		
Depression	0.79	0.72–0.87	<0.001

### Achievement of 2016 European Society of Cardiology/European Atherosclerosis Society lipid guideline targets

Participants who had undergone lipid testing were grouped into those with and without prior depression. The number of participants prescribed a HI statin within 90-days of discharge was similar between the two groups (57.8 vs 58.0% non-depressed vs depressed), but lower in the depression group for low intensity statin (36.0 vs 33.9%) and higher for combination statin+other LLT (1.7 vs 3.4% respectively). However, the odds of receiving a statin prescription immediately post PCI were similar in patients with and without depression (0.85 95%CI 0.68–1.07 p = 0.17).

For all patients with a lipid level record the lowest levels of LDL-C, non-HDL-C and TG achieved in the year post PCI were higher on average in those with depression than those without (2.02 ± 0.83 vs 1.88 ± 0.76 mmol/L, 2.76 ± 1.02 vs 2.51 ± 0.93 mmol/L & 1.58 ± 1.40 vs 1.36 ± 1.33 mmol/L respectively: all p<0.001). No difference in HDL-C levels was seen between those with and without depression (1.15 ± 0.35 vs 1.16 ± 0.34 mmol/L respectively p = 0.64).

#### LDL-C

There were 9,067 participants who had a measured LDL-C level between 28-days and 1-year of discharge. Participants with depression were less likely to meet the ESC/EAS 2016 guideline target of <1.8mmol/L within 1 year of discharge (943 [42.2%] vs 3360 [49.2%] p<0.001 respectively).

In the logistic regression analysis, those with depression were less likely to achieve the 2016 LDL target (OR 0.86 95%CI 0.78–0.96 p = 0.007), (Table 3, with very similar results obtained using DAG model selection, S5A Table).

**Table 3:** Full mutually adjusted binary logistic regression identifying variables associated with achievement of ESC/EAS 2016 LDL-C target (<1.8mmol/L) during first year of follow up post percutaneous coronary intervention.

Covariate	Odds ratio	95% C.I.	p
Age	1.02	1.01–1.02	<0.001
Female	0.55	0.50–0.61	<0.001
Diabetes	1.60	1.44–1.79	<0.001
Contemporary acute coronary syndrome	1.58	1.42–1.76	<0.001
Hypertension	0.99	0.90–1.09	0.91
Chronic kidney disease	1.03	0.63–1.68	0.92
Heart failure	0.89	0.78–1.02	0.10
Ischaemic stroke	0.88	0.73–1.07	0.20
Peripheral vascular disease	0.63	0.52–0.78	<0.001
Atrial fibrillation	0.88	0.76–1.03	0.13
Lipid lower therapy (LLT)			<0.001
No LLT	REF		
High statin	9.37	7.00–12.54	
Low statin	4.00	2.99–5.35	
Other	0.56	0.27–1.18	
Statin + other	3.39	2.16–5.31	
Deprivation index			0.09
1 (most deprived)	1.05	0.91–1.21	
2	0.94	0.82–1.09	
3	0.91	0.79–1.05	
4	1.09	0.94–1.26	
5 (least deprived)	REF		
Depression	0.86	0.78–0.96	0.007

**Fig 1:**
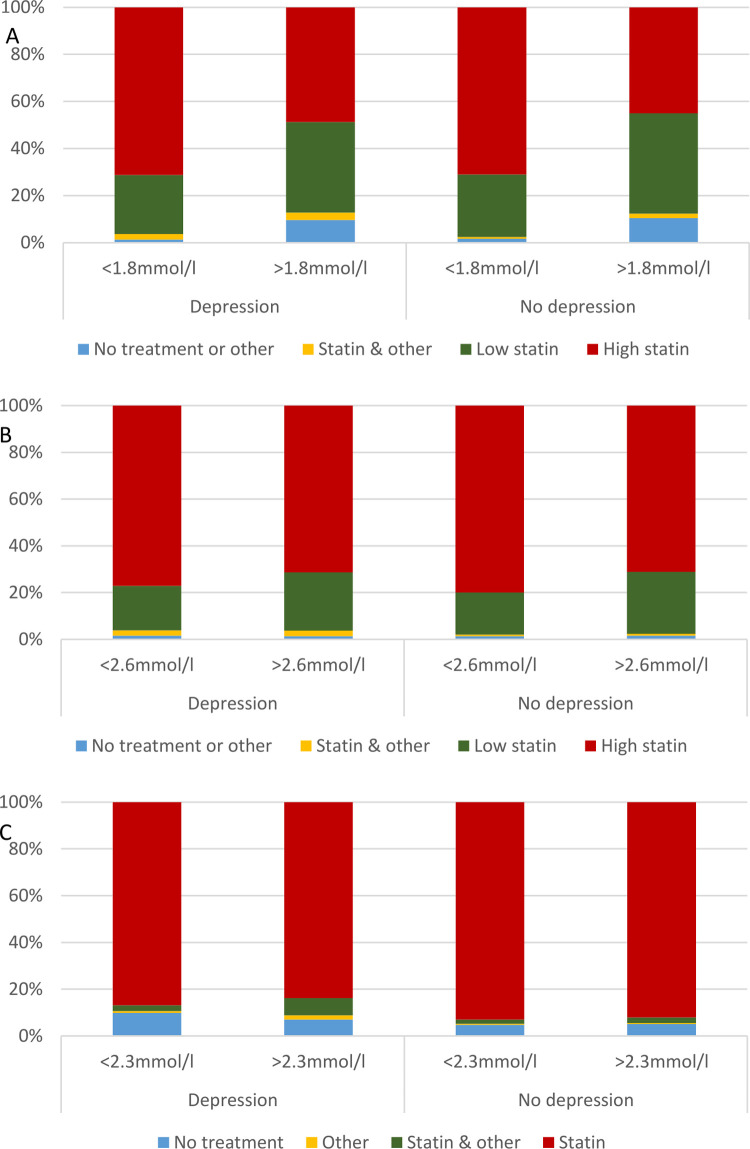
A-C Prescribed lipid lowering therapy regimes in patients meeting and not meeting 2016 European Society of Cardiology/European Atherosclerosis Society guideline lipid targets according to depression status for low-density lipoprotein cholesterol (A), non-high-density lipoprotein cholesterol (B) and triglycerides (C). Other stands for prescription of ezetimibe or fibrate. For Fig C other represents fibrate or N-3 fatty acid.

Patients with depression who did not meet the LDL-C target were less likely to be on LLT than those who achieved the target (Fig 1A) and were less likely to be treated with HI statin than depressed patients who met the lipid target (48.8% vs 71.3%). However, the proportion of depressed and non-depressed patients who did not meet the target treated with HI statin were similar (48.8% vs 45.0%), Fig 1A.

#### Non-HDL-C

Of the 4,837 participants who had a non-HDL-C level, those with depression were less likely to meet the ESC/ESA 2016 target of <2.6 mmol/L within 1-year of discharge, 634 (49.5%) vs 2127 (59.8%) respectively; p<0.001.

In the logistic regression analyses, patients with depression were less likely to meet these guideline targets for non-HDL-C (OR 0.80 95%CI 0.69–0.92 p = 0.003), (Table 4, with very similar results obtained using DAG model selection, S5B Table).

**Table 4:** Full mutually adjusted binary logistic regression identifying variables associated with achievement of ESC/EAS non-HDL-C target (<2.6mmol/L) during first year of follow up post percutaneous coronary intervention.

Covariate	Odds ratio	95% C.I.	p
Age	1.03	1.02–1.04	<0.001
Female	0.63	0.55–0.73	<0.001
Diabetes	1.16	1.00–1.35	0.056
Contemporary acute coronary syndrome	1.55	1.33–1.81	<0.001
Hypertension	0.84	0.73–0.96	0.009
Chronic kidney disease	0.94	0.45–1.98	0.88
Heart failure	0.84	0.69–1.02	0.08
Ischaemic stroke	0.90	0.70–1.17	0.43
Peripheral vascular disease	0.56	0.42–0.75	<0.001
Atrial fibrillation	1.09	0.87–1.37	0.43
Lipid lower therapy (LLT)			<0.001
No LLT	REF		
High statin	13.64	9.20–20.22	
Low statin	5.52	3.71–8.23	
Other	0.47	0.16–1.41	
Statin + other	3.31	1.82–6.02	
Deprivation index			0.07
1 (most deprived)	1.05	0.86–1.27	
2	0.82	0.67–1.00	
3	0.85	0.70–1.04	
4	0.91	0.74–1.12	
5 (least deprived)	REF		
Depression	0.80	0.69–0.92	0.003

Patients with depression who did not achieve the non-HDL-C target were more likely to be treated with HI-statin or combination statin post-PCI compared to those without depression (54.3% vs 51.1% & 4.8% vs 2.4% respectively), Fig 1B. In patients who did meet the non-HDL-C target, those with depression were less likely to be treated with HI statin and more likely to be on other or no LLT than those without depression (Fig 1B).

#### Triglycerides

There were 9,595 patients with documentation of TG level between 28-days and 1-year post-PCI. Most patients met the TG target of <2.3mmol/L (81.8%). However, fewer of those with depression met the TG target than those without depression (1,919 [79.8%] vs 6,302 [87.7%] p<0.001). In the logistic regression analyses, patients with depression were less likely to meet these guideline targets for TG (OR 0.69 95CI% 0.61–0.79 p<0.001) (Table 5, with very similar estimates obtained using the DAG model selection, S5C Table).

**Table 5:** Full mutually adjusted binary logistic regression identifying variables associated with achievement of ESC/EAS 2016 triglyceride target (<2.3mmol/L) during first year of follow up post percutaneous coronary intervention.

Covariate	Odds ratio	95% C.I.	p
Age	1.04	1.03–1.04	<0.001
Female	1.02	0.89–1.18	0.75
Diabetes	0.51	0.44–0.58	<0.001
Contemporary acute coronary syndrome	1.39	1.22–1.59	<0.001
Hypertension	0.74	0.65–0.84	<0.001
Chronic kidney disease	0.70	0.42–1.17	0.17
Heart failure	0.95	0.80–1.13	0.54
Ischaemic stroke	0.73	0.58–0.92	0.01
Peripheral vascular disease	0.76	0.60–0.97	0.025
Atrial fibrillation	1.18	0.94–1.48	0.15
Lipid lower therapy (LLT)			<0.001
No LLT	REF		
Fibrate or N3	0.73	0.38–1.40	
Statin	1.97	1.57–2.48	
Statin + other	1.11	0.76–1.64	
Deprivation index			0.07
1 (most deprived)	0.76	0.63–0.92	
2	0.84	0.69–1.03	
3	0.82	0.68–1.00	
4	0.87	0.71–1.08	
5 (least deprived)	REF		
Depression	0.69	0.61–0.79	<0.001

Patients with depression who did not meet the guideline level were less likely to be on statin therapy (83.8 vs 86.9%) but more likely to be on statin combination LLT (7.4% vs 2.5%) or fibrates and/or N3 (1.8 vs 0.8%) than non-depressed patients (Fig 1C). Additionally, patients with depression who met the TG target were more likely to be treated with statin therapy and less likely to be prescribed other LLT than depressed patients who did not meet the target (Fig 1C).

#### 2019 ESC/EAS lipid guideline targets

The proportion of patients meeting the 2019 ESC/EAS LDL-C, non-HDL-C and TG targets was also explored. Within the whole cohort, fewer people met the 2019 than the 2016 targets for all lipid subtypes (LDL-C 2016 47.5% vs 2019 22.7%, non-HDL-C 57.1 vs 36.8% & TG 81.8 vs 59.2%). However, as we found with 2016 targets, those with depression were less likely to have achieved 2019 targets than those without (LDL-C 19.7 vs 23.7%, non-HDL-C 29.2 vs 39.6% & TG 50.1 vs 62.2% respectively; all p<0.001).

Multiple regression analyses identified depression as being associated with lower odds of meeting 2019 targets for both non-HDL-C and TG (in both full adjusted model and DAG model selection, S6 and S7 Tables).

#### Sex and depression

**Fig 2:**
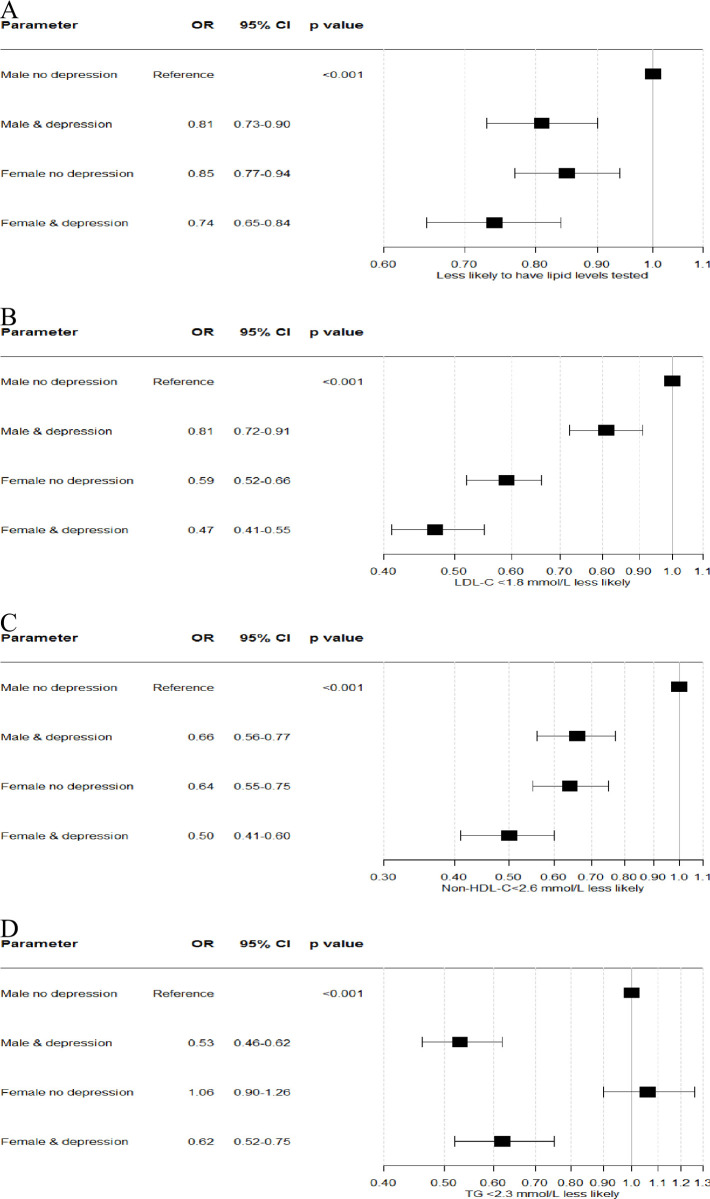
Relationship between sex and depression and proportion of population having a A) documented lipid level or achieving 2016 ESC/EAS lipid guideline targets for B) LDL-C, C) non-HDL-C and D) triglycerides.

Females were less likely to achieve the 2016 and 2019 LDL-C and non-HDL-C and 2019 TG lipid targets in all multivariable models which adjusted for clinical variables, depression and socioeconomic status (Tables 3 and 4 & S6 Table, and with very similar estimates from relevant DAG guided model selection (S7 Table)). Although a significant interaction between sex and depression was not found on formal testing, an important cumulative effect of female sex together with a prior diagnosis of depression was identified with regards to both the odds of undergoing lipid testing and achieving LDL-C and non-HDL targets (Fig 2 and S2 Fig).

In those patients who had both pre and post lipid measurements (LDL-C n = 4896, non-HDL-C n = 1789 & TG n = 5480), the percentage reduction was calculated. There was a non-significant trend for those with depression to have a smaller reduction in LDL-C and non-HDL-C levels. In a pairwise comparison women with depression had a significantly smaller percentage reduction in LDL-C than non-depressed males (p = 0.034 S3 Fig).

## Discussion

In this study of real-world data looking at the management of lipid levels in patients who had undergone PCI, those with prior depression were found not only to be less likely to have a lipid level test but also less likely to achieve the ESC/EAS guideline target levels for LDL-C, non-HDL-C and TG if tested within a year post-PCI. Furthermore, even after accounting for their greater prevalence of depression and adjusting for clinical and socioeconomic status (deprivation index), female sex remained independently associated with less testing and effective control of lipids. Thus, women with prior depression were the least likely group to undergo lipid testing and to meet guideline targets when tested. This work has focused on the clinical management of lipids in these very high-risk patients in the setting of increasingly aggressive International guideline targets, in order to identify potential areas for improvement in care rather than evaluating associations between lipids and depression.

The 2016 and 2019 ESC/EAS guideline targets were both considered in this study. Although most patients underwent their PCI prior to publication of these guidelines, the 2016 LDL target for this population is the same as the 2012 ESC guidelines [16]. Importantly, these very high-risk, patients qualify for the newer, more aggressive targets set out in the most recent guidelines, which is illustrating the challenges of lipid management in this very high-risk patient population–particularly those with depression. A similar approach to analysis of HDL-C levels was not undertaken in this study as HDL-C modifying therapies have not been associated with improvements in outcomes and HDL-C treatment targets are not specified in the guidelines [15]. Although, HDL-C levels are important for calculating CVD risk in primary prevention, the ESC/EAS guideline do not currently require this information for classification of CVD risk and approach to lipid management in patients with established atherosclerotic coronary artery disease.

There is limited work in this area but the finding that patients with depression were less likely to meet LDL-C targets agrees with Katzmann et al., who also showed that depressed patients were less likely to adhere to their medication [6]. A 2012 study has also shown that patients with depression identified post cardiac event, were less likely to achieve target lipid levels [17]. Our findings of less testing and achievement of targets in those checked, is consistent with findings in patients with severe mental illness, who are less likely to receive annual CV screening, have delayed identification and treatment of risk factors, which when treated, are treated less intensively [4, 5, 18]. Whether these important issues are being overlooked due to clinicians prioritising mental health and other attendant problems cannot be determined in a study of this nature using routinely held EHR clinical data. However, it is worth noting that patients with diabetes were more likely to have their lipid levels checked post-PCI, probably due to this being a recommendation in the diabetes and CVD clinical care pathways, which could be a consideration for other important co-morbidities such as depression. A better understanding of the determinants of these inequities that may contribute to adverse outcomes would require a prospective clinical study. In this study we were not able to determine whether a lack of lipid test was due to it not being offered to the patients or their failure to attend for testing. Future work would look to explore both number and types of interactions with healthcare providers to see whether this could provide insights as to why some patients did not have their lipid levels tested.

Despite being at increased risk of initially developing CVD and further events in those already with coronary heart disease, the relationship between dyslipidaemia and depression is not clear cut [1, 2]. Studies have shown depressed patients are more likely to have greater LDL-C and TG’s, and lower HDL-C, alongside obesity [19, 20]. This metabolic profile may suggest some influence of lifestyle factors and psychophysiological stress, which may be particularly pertinent regarding TG’s and TG rich lipoproteins [21]. Elevated TG’s, particularly in the Western world, are commonly associated with a diet high in refined and processed foods and alcohol consumption, thus lifestyle modification could be the most effective method for TG reduction in those with depression, although pharmaceutical agents are available for those with very high or persistently elevated levels [22]. Conversely, a meta-analysis has suggested that patients with depression have lower LDL [23]. Nonetheless, in our study we found that patients with depression had higher levels of LDL-C, non-HDL-C and TG than those without depression post PCI and that a greater proportion were not at target levels, suggesting increased risk of further cardiovascular events.

The finding that females with depression were the least likely to be tested and achieve LDL-C and non-HDL-C targets is to some extent unsurprising, given what is already known from studies examining relationships between depression or sex and CVD outcomes. However, our observation of a substantial additive effect on the quality of lipid management in a very high CVD risk population is a novel and important finding, identifying a group of patients who could benefit substantially from improved preventive care. Despite improvements in care and treatment of CVD the decline in mortality from coronary heart disease has been greater in men than women [24]. Differences in the presentation of coronary artery disease can lead to delays in diagnosis in women, whilst women at high risk or with established CVD are less likely to be prescribed statins in primary care [25]. Women have also been under-represented in clinical trials to test lipid lowering therapy and are less likely to achieve lipid target levels. These areas and the other literature looking at optimisation of lipid management in women have recently been reviewed by Peterson et al. [26]. Considering together the greater prevalence of depression in women and likelihood of reduced adherence to medication in those with depression, our findings highlight an important area in the field of CVD prevention that merits further research to understand and manage better these identified gaps in quality of clinical care [9, 27]. This should result in improved outcomes in these high-risk patients. Notably, this relationship was not seen with achievement of TG targets, which is likely due to men generally having greater TG levels than women, and treatment of moderate hypertriglyceridaemia not currently being endorsed by the UK National Institute for Health and Care Excellence guidance [28]. Interestingly, depressed women were still less likely to reach the 2019 ESC/EAS TG target which may imply that TG levels are not being treated intensively enough in women either through medication or lifestyle advice and modification. The lack of a significant association between the percentage change in LDL-C or non-HDL-C levels pre and post PCI with sex and/or depression should not detract from the finding that those with depression had higher lipid levels and were less likely to achieve guideline targets post PCI, particularly in women. The number of patients with available data for both pre and post-PCI lipid levels was limited particularly for non-HDL-C and may not be representative of the whole study population.

Although there were some differences in the prescribing of lipid lowering therapy in those with and without depression, it would appear that the medication regimes were at least as good in depressed patients as in the non-depressed. Depressed patients were proportionally more likely to be on ezetimibe and or fibrate with a statin than non-depressed, but the numbers on these treatments and particularly those without a statin were too small to explore the reasons for this further. Despite these differences in medications, depressed patients were less likely to reach target levels. Other possible explanations, which we were unable to explore in this study using a routinely held clinical dataset, may include reduced medication concordance and impaired response to medication in depressed patients undergoing PCI. Indeed, a previous meta-analysis has shown that depressed patients were less likely to comply with medical treatment for non-cardiovascular conditions [27]. Prospective studies including prescribing and dispensing data as well as lipid levels would be required to investigate concordance and responses to medication more fully. Additionally, investigating the impact of PCSK-9 inhibitors on the achievement of lipid targets would be helpful. This was not possible in the current study as these were only approved for use within the UK National Health Service in June 2016, were only prescribed through specialist hospital outpatient clinics during the study period (from which data were not available) and uptake has also been low in Wales [29, 30]. Therefore, no patients were identified as being prescribed PCSK-9 inhibiting drugs in our study.

Why depression results in an increased risk of CVD is not fully understood but is likely to be due to both biological and social factors. Biological causes such as increased hypothalamus-pituitary-adrenal activity, endothelial dysfunction and inflammatory processes are impacted by social factors such as increased alcohol intake, smoking, physical inactivity and poor diet [20, 31–33]. Indeed, in this study, patients with depression were more likely to be obese or current smokers than those without depression, which could contribute to their increased risk and, in the case of obesity, failure to achieve lipid targets. Those with depression are commonly more adversely affected by socioeconomic factors which impact on other lifestyle and physiological contributors to cardiometabolic risk. It is worth noting that in this study socioeconomic status (as measured by deprivation index) was only weakly associated with assessment of lipid levels and not with achievement of lipid targets after accounting for clinical variables, sex and depression. Despite this and although difficult, measures to reduce socio-economic inequalities and encouragement of patients to modify these factors may help to improve their lipid and other risk factor profile. Specifically, ensuring prescription of and adherence to appropriate medication, as well as monitoring of effectiveness of treatment are potentially simple and effective measures to address these inequalities. The ESC/EAS guidelines recommend prescription of more potent doses of statins, where appropriate and tolerated, and/or consideration of newer treatments such as anti-PCSK-9 therapies [15]. The latter have a longer half-life and are likely to be more successful at achieving targets in those patients with lower tolerance of and/or concordance with statin therapy, or levels of LDL-C and HDL-C that remain well above target levels despite statin therapy [15]. Together these measures may help to reduce the imbalance in risk of CVD between patients with and without depression, especially in women.

It is noteworthy that some patients who might be expected to have regular lipid monitoring such as those with chronic kidney or peripheral vascular disease, were shown to be less likely to receive checks in our study. The exact reasons for this cannot be determined in a study of this nature, but may be due to the risk-treatment paradox where high-risk patients are treated less intensively due to a perceived risk of treatment complications or side effects.

Although we cannot be confident of all the reasons for why patients prescribed lipid lower therapy were more likely to be tested, it was not a surprising finding, as medical professionals are more likely to monitor the impact of treatments they prescribe and make adjustments according to clinical responses. This only heightens the importance of our observation that those not being prescribed LLT are not having their lipids checked nor being treated as effectively as those already receiving treatment.

### Limitations

There are some limitations to this work. We were only able to look at lowest lipid level achieved within the time frame and not percentage reduction in the whole population as not all patients had a lipid profile recorded at admission. This may have identified a larger proportion of patients who did not meet target reductions. The time period (28–365 days) for having lipids checked and achieving lowest lipid level was relatively broad but this was to allow a reasonable time period for assessment and management of lipid levels in routine practice over the important first year post-PCI. Unfortunately, a study of this nature does not permit assessment of medication concordance or adherence, as we only had access to prescribing data, nor were data on medication intolerance or attention to lifestyle modification available, as this information is not currently reliably captured in electronic health records. More comprehensive documentation of these factors would be necessary to understand better why patients were not meeting targets and whether some or all of these had a greater influence in those with depression.

Not all those prescribed antidepressants had an associated depression diagnosis or symptom [12, 34]. This could be for a variety of reasons as they are often prescribed to patients with pain, cancer and numerous other conditions. Patients prescribed an antidepressant were not categorised as having had depression in our study if they did not have a corresponding diagnostic code for depression diagnosis or symptoms in their primary care record. It may therefore be the case that some patients were miscategorised if an appropriate diagnostic code was not entered by their GP. However, we lack the numbers in the current study to effectively look at this.

Whilst we excluded those with anxiety (without depression) and severe mental illness, adjustment was not made for those with other psychiatric co-morbidities. Such co-morbidities are common across a wide range of psychiatric disorders and future work will explore these in more depth adjusting for both the number of co-morbidities and by co-morbidity.

In this analysis we have only been able to examine differences in lipid testing, treatment and control according to the individuals’ documented sex. Although biological sex is likely to have an important influence on these outcomes, it is also probable that gender characteristics could play an important role. For example, Pelletier et al. have noted that feminine gender characteristics were more strongly associated with adverse outcomes in patients with premature coronary artery disease [35]. It was not possible to explore these issues in any detail beyond the documented sex of the individuals assessed in this anonymised, routinely-held clinical dataset. However, it will be important to ensure that the relative contribution of sex and gender on relevant clinical outcomes can be determined in further prospective studies in this area.

In conclusion, we have demonstrated in a large high-risk group of patients who underwent PCI, that those with depression were not only less likely to have their lipid levels tested post-PCI, but also even if checked were less likely to achieve ESC/EAS lipid guideline targets. Strikingly, females with depression were by far the least likely to be tested and achieve targets. Our observations have identified large groups of patients who might benefit further from greater attention being paid to their physical as well as mental health. Specifically, we have identified opportunities to improve lipid management and therefore potentially clinical outcomes through measures addressing lifestyle optimisation, improved medication concordance and prescription of additional evidence-based medications where appropriate.

## Supporting information

S1 File Directed acyclic graph model selection. (DOCX)

S1 Fig Study population cohort selection. (TIF)

S2 Fig Relationship between sex and depression and odds of achieving 2019 ESC/EAS lipid guideline targets for (A) LDL-C, (B) non-HDL-C and (C) Triglycerides.
(DOCX)

S3 Fig Percentage change between pre and post percutaneous levels of (A) LDL-C, (B) non-HDL-C and (C) Triglycerides. Number of patients with both pre and post levels for (A) LDL-C = 4896, (B) non-HDL-C = 1789 and (C) Triglycerides = 5480.
(DOCX)

S1 Table Diagnostic (Read) codes for depression, mixed anxiety/depression, anxiety and severe mental illness. (DOCX)

S2 Table Variables associated with being prescribed statin therapy pre-percutaneous coronary intervention. Estimates for age are per year.
(DOCX)

S3 Table Patient characteristics for the whole cohort and comparison between those with and without a documented lipid level during follow-up. ACS: acute coronary syndrome; LLT: lipid lowering therapy; MI: myocardial infarction; PCI: percutaneous coronary intervention; PVD: peripheral vascular disease; Other includes ezetimibe and/or fibrate.
(DOCX)

S4 Table Directed acyclic graph guided binary logistic regression for estimating the effect of depression on odds of having a documented lipid level during follow-up. (DOCX)

S5 Table Directed acyclic graph guided binary logistic regression estimating the effect of depression on achieving ESC/EAS 2016 (A) low density lipoprotein, (B) non-high-density lipoprotein and (C) triglyceride targets during follow-up.
(DOCX)

S6 Table Full mutually adjusted binary logistic regression identifying variables associated with achieving ESC/EAS 2019 targets during follow-up for (A) LDL-C, (B) non-HDL-C and (C) triglycerides.
(DOCX)

S7 Table Directed acyclic graph guided binary logistic regression for estimating the effect of depression on achieving ESC/EAS 2019 (A) LDL-C, (B) non-HDL-C and (C) triglyceride targets during follow-up.
(DOCX)
