# Supplementary material for: Achievement of European Society of Cardiology/European Atherosclerosis Society lipid targets in very high-risk patients: Influence of depression and sex
Source: PLoS One. 2022 Feb 25;17(2):e0264529. doi: 10.1371/journal.pone.0264529 (PMC8880762; doi:10.1371/journal.pone.0264529)
Supplement: S1 File — (DOCX) [file pone.0264529.s001.docx]

Supplement 1 Directed Acyclic Graph Model Selection

In the main text we estimate the effect of depression on monitoring and target achievement after adjustment for a wide range of covariates (tables 2 to 5). Due to the potential for inter dependencies between several variables in this data, and hence the possibility of over- and or unnecessary adjustment (through mediator or collider effects), we explored the sensitivity of our main output (odds ratios for depression) to model selection options. We compared the ‘full’ model to a minimally sufficient model (for the effect of depression on the outcome) obtained from assumed prior directed acyclic graphs (DAGs) for each outcome. The relationships to build the DAGs were guided by the clinical knowledge of the investigators. Assumed DAGs and results are given below. We note that, in each case, the estimate of the odds ratio for the covariate depression (and, where relevant, sex), was not sensitive to use of the full covariates or the minimally sufficient set.


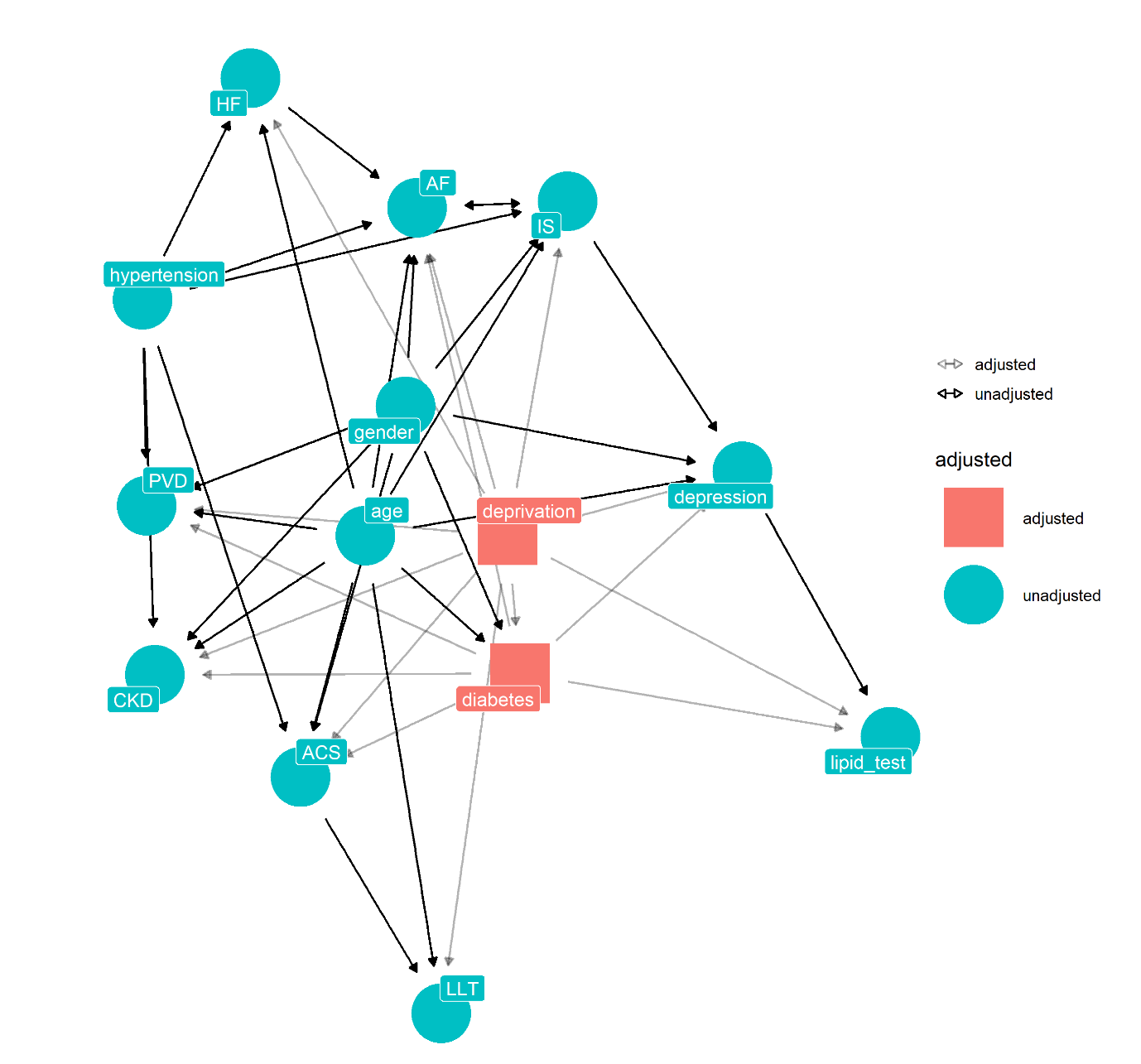


Supplementary Figure 1: Directed acyclic graphs for covariate selection for multivariate analyses exploring association of depression and odds of lipid level assessment or achievement of ESC/EAS 2016 and 2019. The DAG shows the relationships between study variables. Those in pink are suggested by the DAG for adjustment to provide the least biased estimate of the relationship between depression and lipid test.


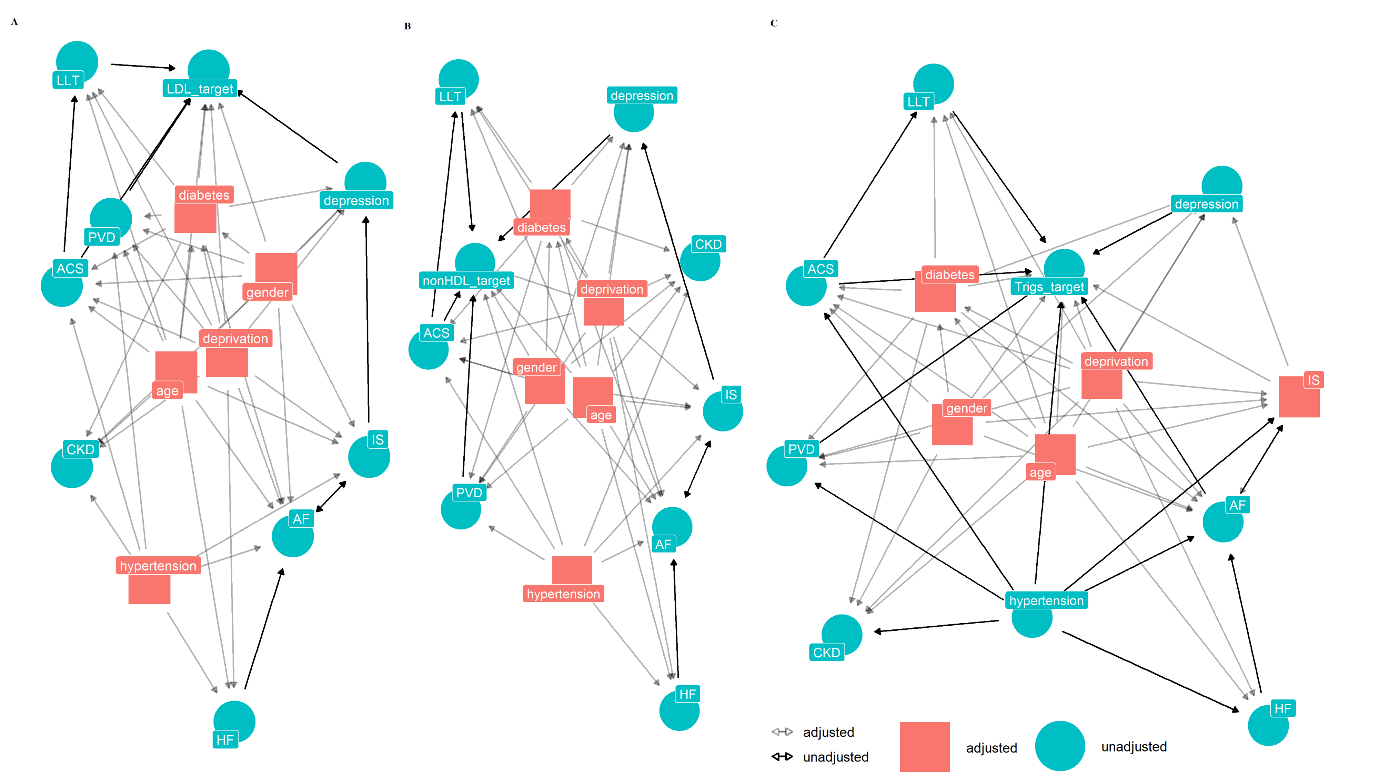


Supplementary Figure 1: Directed acyclic graphs for covariate selection for multivariate analyses exploring association of depression and odds of (A) LDL-C, (B) non-HDL-C and (C) triglyceride targets during follow-up. ACS: acute coronary syndrome; AF: atrial fibrillation; CKD: chronic kidney disease; HF: heart failure; IS: ischaemic stroke; LLT: lipid lowering therapy; PVD: peripheral vascular disease.
