# Supplementary material for: Achievement of European Society of Cardiology/European Atherosclerosis Society lipid targets in very high-risk patients: Influence of depression and sex
Source: PLoS One. 2022 Feb 25;17(2):e0264529. doi: 10.1371/journal.pone.0264529 (PMC8880762; doi:10.1371/journal.pone.0264529)
Supplement: S2 Fig — Relationship between sex and depression and odds of achieving 2019 ESC/EAS lipid guideline targets for (A) LDL-C, (B) non-HDL-C and (C) Triglycerides. (DOCX) [file pone.0264529.s003.docx]

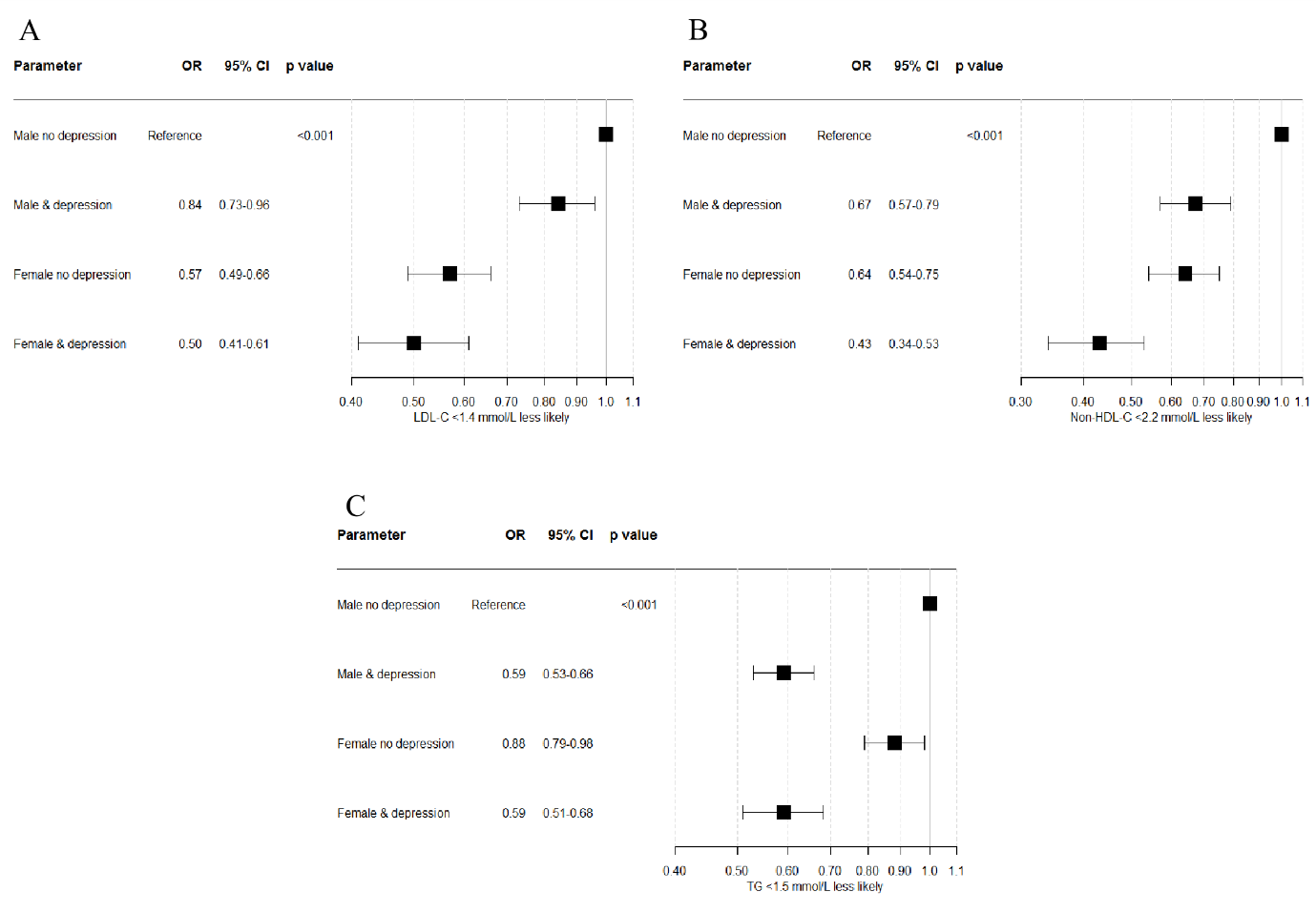


**S2 Fig.** **Relationship between sex and depression and odds of achieving 2019 ESC/EAS lipid guideline targets for (A) LDL-C, (B) non-HDL-C and (C) Triglycerides.**
