# Supplementary material for: Achievement of European Society of Cardiology/European Atherosclerosis Society lipid targets in very high-risk patients: Influence of depression and sex
Source: PLoS One. 2022 Feb 25;17(2):e0264529. doi: 10.1371/journal.pone.0264529 (PMC8880762; doi:10.1371/journal.pone.0264529)
Supplement: S3 Fig — Percentage change between pre and post percutaneous levels of (A) LDL-C, (B) non-HDL-C and (C) Triglycerides. Number of patients with both pre and post levels for (A) LDL-C = 4896, (B) non-HDL-C = 1789 and (C) Triglycerides = 5480. (DOCX) [file pone.0264529.s004.docx]

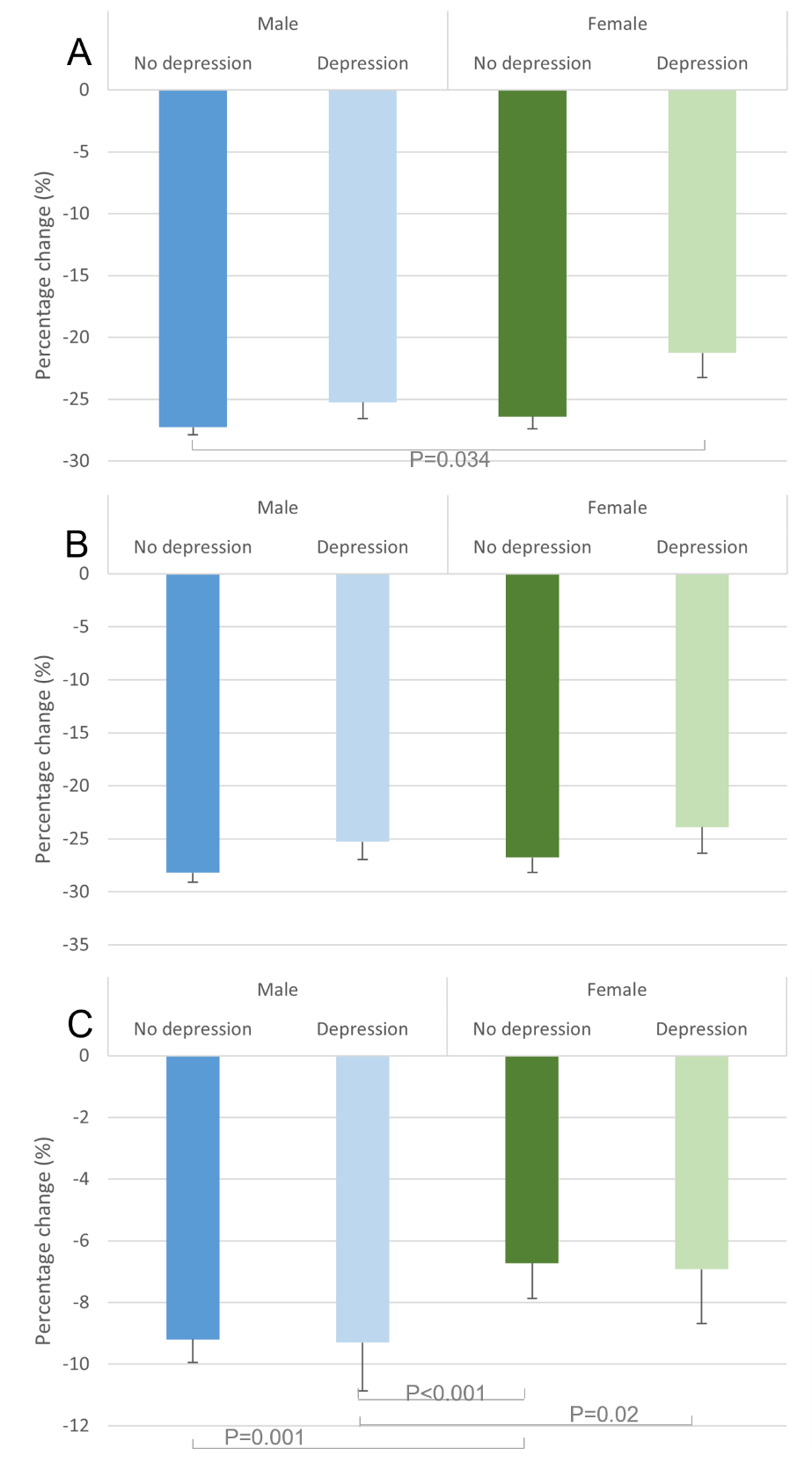


**S3 Fig. Percentage change between pre and post percutaneous levels of (A) LDL-C, (B) non-HDL-C and (C) Triglycerides.** Number of patients with both pre and post levels for (A) LDL-C = 4896, (B) non-HDL-C = 1789 and (C) Triglycerides = 5480.
