# Supplementary material for: Achievement of European Society of Cardiology/European Atherosclerosis Society lipid targets in very high-risk patients: Influence of depression and sex
Source: PLoS One. 2022 Feb 25;17(2):e0264529. doi: 10.1371/journal.pone.0264529 (PMC8880762; doi:10.1371/journal.pone.0264529)
Supplement: S1 Table — (DOCX) [file pone.0264529.s005.docx]

**Table 1. Diagnostic (Read) codes for depression, mixed anxiety/depression, anxiety and severe mental illness.**

|  | **Read code** | **Description** |
| --- | --- | --- |
| **Depression diagnosis** | E112. | single major depressive episode |
|  | E1120 | single major depressive episode, unspecified |
|  | E1121 | single major depressive episode, mild |
|  | E1122 | single major depressive episode, moderate |
|  | E1123 | single major depressive episode, severe, without psychosis \| single major depressive episode, severe, without mention of psychosis |
|  | E1125 | single major depressive episode, in partial or unspecified remission |
|  | E1126 | single major depressive episode, in full remission |
|  | E112z | single major depressive episode nos |
|  | E113. | recurrent major depressive episode |
|  | E1130 | recurrent major depressive episodes, unspecified |
|  | E1131 | recurrent major depressive episodes, mild |
|  | E1132 | recurrent major depressive episodes, moderate |
|  | E1133 | recurrent major depressive episodes, severe, without mention of psychosis |
|  | E1135 | recurrent major depressive episodes, in partial or unspecified remission |
|  | E1136 | recurrent major depressive episodes, in full remission |
|  | E1137 | recurrent depression |
|  | E113z | \| recurrent major depressive episode nos |
|  | E118. | seasonal affective disorder |
|  | E135. | agitated depression |
|  | E204. | neurotic depression reactive type |
|  | E291. | prolonged depressive reaction |
|  | E2B.. | depressive disorder nec |
|  | E2B0. | postviral depression |
|  | E2B1. | chronic depression |
|  | Eu32. | [x]depressive episode |
|  | Eu320 | [x]mild depressive episode |
|  | Eu321 | [x]moderate depressive episode |
|  | Eu322 | [x]severe depressive episode without psychotic symptoms |
|  | Eu324 | [x]mild depression |
|  | Eu325 | [x]major depression, mild |
|  | Eu326 | [x]major depression, moderately severe |
|  | Eu327 | [x]major depression, severe without psychotic symptoms |
|  | Eu32B | [x]antenatal depression |
|  | Eu32y | [x]other depressive episodes |
|  | Eu32z | [x]depressive episode, unspecified |
|  | Eu33. | [x]recurrent depressive disorder |
|  | Eu330 | [x]recurrent depressive disorder, current episode mild |
|  | Eu331 | [x]recurrent depressive disorder, current episode moderate |
|  | Eu332 | [x]recurrent depressive disorder, current episode severe without psychotic symptoms |
|  | Eu334 | [x]recurrent depressive disorder, currently in remission |
|  | Eu33y | [x]other recurrent depressive disorders |
|  | Eu33z | [x]recurrent depressive disorder, unspecified |
|  | Eu341 | [x]dysthymia |
|  | 9HA0. | On depression register |
|  | 62T1. | Puerperal depression |
|  | 1465 | history of depression |
|  |  |  |
| **Depression symptoms** | 1B17. | depressed |
|  | 1B1U. | symptoms of depression |
|  | 1BP.. | loss of interest |
|  | 1BP0. | loss of interest in previously enjoyable activity |
|  | 1BQ.. | loss of capacity for enjoyment |
|  | 1BT.. | depressed mood |
|  | 1BU.. | loss of hope for the future |
|  | 2257. | o/e - depressed |
|  |  |  |
| **Mixed anxiety/depression** | E2003 | anxiety with depression |
|  | Eu412 | [x]mixed anxiety and depressive disorder |
|  |  |  |
| **Treatment** | 9k4.. | Depression - enhanced service administration |
|  | 9Ov2. | Depression monitoring third letter |
|  | 9k40. | Depression - enhanced services completed |
|  | 9OV.. | Depression monitoring administration |
|  | 8BK0. | Depression management program |
|  | 9hC.. | Exception reporting: depression quality indicators |
|  | 9Ov3. | Depression monitoring verbal invite |
|  | 9Ov4. | Depression monitoring telephone invite |
|  | 9Ov1. | Depression monitoring second letter |
|  | 9kQ.. | On full dose long term treatment depression - enh serv admin |
|  | 8CAa. | Patient given advice about management of depression |
|  | 9Ov0. | Depression monitoring first letter |
|  | 9hC1. | Expected from depression quality indicators: Informed dissen |
|  | TJ90. | Adverse reaction to antidepressants |
|  | TJ90Z | Adverse reaction to antidepressants NOS |
|  | U6092 | [x] Adverse reaction to antidepressant\| [x] Adverse reaction to antidepressants NOS |
|  | 9H92. | Depression interim review |
|  | 9H90. | Depression annual review |
|  | 9H91. | Depression medication review |
|  |  |  |
| **Antidepressants** | d71.. | Amitriptyline hydrochloride |
|  | d72.. | Butriptyline - discontinued |
|  | d73.. | Clomipramine hydrochloride |
|  | d74.. | Desipramine hydrochloride |
|  | d75.. | Dosulepin Hydrochloride |
|  | d76.. | Doxepin |
|  | d77.. | Imipramine hydrochloride |
|  | d78.. | Iprindole |
|  | d79.. | Lofepramine |
|  | d7a.. | Maprotiline hydrochloride |
|  | d7b.. | Mianserin hydrochloride |
|  | d7c.. | Nortriptyline |
|  | d7d.. | Protriptyline hydrochloride |
|  | d7e.. | Trazadone hydrochloride |
|  | d7f.. | Trimipramine |
|  | d7g.. | Viloxazine hydrochloride |
|  | d7h.. | Amoxapine |
|  | d81.. | Phenelzine |
|  | d83.. | Isocarboxazid |
|  | d84.. | Tranylcypromine |
|  | d85.. | Moclobemide |
|  | d91.. | Compound Antidepressants A-Z |
|  | da1.. | Flupentixol [Antidepressant] |
|  | da2.. | Tryptophan |
|  | da3.. | Fluvoxamine Maleate |
|  | da4.. | Fluoxetine hydrochloride |
|  | da5.. | Sertraline hydrochloride |
|  | da6.. | Paroxetine hydrochloride |
|  | da7.. | Venlafaxine |
|  | da9.. | Citalopram |
|  | daA.. | Reboxetine |
|  | daB.. | Mirtazapine |
|  | daC.. | Escitalopram |
|  | daD.. | Agomelatine |
|  | gde.. | Duloxetine |
|  |  |  |
| **Anxiety diagnosis** | E200. | anxiety states |
|  | E2000 | anxiety state unspecified |
|  | E2001 | panic disorder |
|  | E2002 | generalised anxiety disorder |
|  | E2004 | chronic anxiety |
|  | E2005 | recurrent anxiety |
|  | E200z | anxiety state nos |
|  | E202. | phobic disorders |
|  | E2020 | phobia unspecified |
|  | E2021 | agoraphobia with panic attacks |
|  | E2022 | agoraphobia without mention of panic attacks |
|  | E2023 | social phobia, fear of eating in public |
|  | E2024 | social phobia, fear of public speaking |
|  | E2025 | social phobia, fear of public washing |
|  | E2026 | acrophobia |
|  | E2027 | animal phobia |
|  | E2028 | claustrophobia |
|  | E2029 | fear of crowds |
|  | E202A | fear of flying |
|  | E202B | cancer phobia |
|  | E202C | dental phobia |
|  | E202D | fear of death |
|  | E202E | fear of pregnancy |
|  | E202z | phobic disorder nos |
|  | E2D0. | disturbance of anxiety and fearfulness in childhood and adolescence |
|  | E2D00 | childhood and adolescent overanxiousness disturbance |
|  | E2D01 | childhood and adolescent fearfulness disturbance |
|  | E2D0z | disturbance of anxiety and fearfulness in childhood and adolescence nos |
|  | Eu40. | [x]phobic anxiety disorders |
|  | Eu400 | [x]agoraphobia |
|  | Eu401 | [x]social phobias |
|  | Eu402 | [x]specific (isolated) phobias |
|  | Eu403 | [x]needle phobia |
|  | Eu40y | [x]other phobic anxiety disorders |
|  | Eu40z | [x]phobic anxiety disorder, unspecified |
|  | Eu41. | [x] other anxiety disorder |
|  | Eu410 | [x]panic disorder [episodic paroxysmal anxiety] |
|  | Eu411 | [x]generalized anxiety disorder |
|  | Eu413 | [x]other mixed anxiety disorders |
|  | Eu41y | [x]other specified anxiety disorders |
|  | Eu41z | [x]anxiety disorder, unspecified |
|  | Eu930 | [x]separation anxiety disorder of childhood |
|  | Eu931 | [x]phobic anxiety disorder of childhood |
|  | Eu932 | [x]social anxiety disorder of childhood |
|  |  |  |
| **Anxiety symptoms** | 1B12. | nerves - nervousness |
|  | 1B13. | anxiousness |
|  | 1B1V. | c/o - panic attack |
|  | 2258. | o/e - anxious |
|  | 2259. | o/e - nervous |
|  | 225J. | o/e - panic attack |
|  | R2y2. | [d]nervousness |
|  |  |  |
| **Severe mental illness** | E1y.. | other specified non-organic psychoses |
|  | E1z.. | non-organic psychosis nos |
|  | E10% | schizophrenic disorders |
|  | E110% | manic disorder, single episode |
|  | E111% | recurrent manic episodes |
|  | E1124 | single major depressive episode, severe, with psychosis |
|  | E1134 | recurrent major depressive episodes, severe, with psychosis |
|  | E114% | bipolar affective disorder, currently manic |
|  | E115% | bipolar affective disorder, currently depressed |
|  | E116% | mixed bipolar affective disorder |
|  | E117% | unspecified bipolar affective disorder |
|  | E11y. | other and unspecified manic-depressive psychoses |
|  | E11y0 | unspecified manic-depressive psychoses |
|  | E11y1 | atypical manic disorder |
|  | E11y3 | other mixed manic-depressive psychoses |
|  | E11yz | other and unspecified manic-depressive psychoses NOS |
|  | E11z. | other and unspecified affective psychoses |
|  | E11z0 | unspecified affective psychoses NOS |
|  | E11zz | other affective psychotic disorders NOS |
|  | E12% | paranoid states |
|  | E13.. | other nonorganic psychoses |
|  | E130. | reactive depressive psychotic disorders |
|  | E131. | acute hysterical psychotic disorders |
|  | E132. | reactive confusion |
|  | E133. | acute paranoid reaction |
|  | E134. | psychogenic paranoid psychotic disorders |
|  | E13y. | other reactive psychoses |
|  | E13y0 | psychogenic stupor |
|  | E13y1 | brief reactive psychotic disorders |
|  | E13yz | other reactive psychoses NOS |
|  | E13z. | nonorganic psychotic disorders NOS |
|  | E2122 | schizotypal personality |
|  | Eu20% | schizophrenia |
|  | Eu21. | schizotypal disorder |
|  | Eu22% | persistent delusional disorders |
|  | Eu23. | acute and psychotic transient disorders |
|  | Eu231 | acute polymorphic psychotic disorder with symptoms of schizophrenia |
|  | Eu232 | acute schizophrenia like psychotic disorder |
|  | Eu233 | other acute predominantly delusional disorders |
|  | Eu24. | induced delusional disorder |
|  | Eu25% | schizoaffective disorders |
|  | Eu26. | nonorganic psychosis in remission |
|  | Eu2y. | other nonorganic psychotic disorders |
|  | Eu2z. | unspecified non-organic psychosis |
|  | Eu30% | manic episode |
|  | Eu31% | bipolar affective disorder |
|  | Eu323 | severe depressive episode with psychotic symptoms |
|  | Eu328 | major depression, severe with psychotic symptoms |
|  | Eu329 | single major depressive episode, severe, with psychosis, psychosis in remission |
|  | Eu32A | recurrent major depressive episodes, severe, with psychosis, psychosis in remission |
|  | Eu333 | recurrent depressive disorder, current episode severe with psychotic symptoms |
