# Supplementary material for: Achievement of European Society of Cardiology/European Atherosclerosis Society lipid targets in very high-risk patients: Influence of depression and sex
Source: PLoS One. 2022 Feb 25;17(2):e0264529. doi: 10.1371/journal.pone.0264529 (PMC8880762; doi:10.1371/journal.pone.0264529)
Supplement: S2 Table — Estimates for age are per year. (DOCX) [file pone.0264529.s006.docx]

**S2 Table. Variables associated with being prescribed statin therapy pre-percutaneous coronary intervention.**

| **Covariate** | **Odds ratio** | **95% C.I.** | **p** |
| --- | --- | --- | --- |
| Age | 1.02 | 1.01-1.02 | <0.001 |
| Female | 0.78 | 0.71-0.85 | <0.001 |
| Diabetes | 2.57 | 2.34-2.82 | <0.001 |
| Hypertension | 1.93 | 1.79-2.09 | <0.001 |
| Previous myocardial infarction | 1.72 | 1.54-1.93 | <0.001 |
| Previous revascularisation | 4.42 | 3.73-5.23 | <0.001 |
| Chronic kidney disease | 0.76 | 0.54-1.08 | 0.13 |
| Heart failure | 0.98 | 0.87-1.09 | 0.70 |
| Ischaemic stroke | 2.21 | 1.87-2.62 | <0.001 |
| Peripheral vascular disease | 1.98 | 1.67-2.35 | <0.001 |
| Atrial fibrillation | 1.09 | 0.96-1.24 | 0.20 |
| Deprivation index |  |  | 0.007 |
| 1 (most deprived) | 1.13 | 1.00-1.27 |  |
| 2 | 1.02 | 0.90-1.14 |  |
| 3 | 0.93 | 0.83-1.05 |  |
| 4 | 0.92 | 0.82-1.04 |  |
| 5 (least deprived) | REF |  |  |
| Depression | 1.21 | 1.10-1.32 | <0.001 |

estimates for age are per year.
