# Supplementary material for: Achievement of European Society of Cardiology/European Atherosclerosis Society lipid targets in very high-risk patients: Influence of depression and sex
Source: PLoS One. 2022 Feb 25;17(2):e0264529. doi: 10.1371/journal.pone.0264529 (PMC8880762; doi:10.1371/journal.pone.0264529)
Supplement: S3 Table — ACS: acute coronary syndrome; LLT: lipid lowering therapy; MI: myocardial infarction; PCI: percutaneous coronary intervention; PVD: peripheral vascular disease; Other includes ezetimibe and/or fibrate. (DOCX) [file pone.0264529.s007.docx]

**S3 Table. Patient characteristics for the whole cohort and comparison between those with and without a documented lipid level during follow-up.**

|  | **Total cohort n=13,781** | **With lipids**  **n= 10,050** | **Without lipids n=3731** | **p** |
| --- | --- | --- | --- | --- |
| Percentage of cohort (%) | 100 | 72.9 | 27.1 |  |
| Age (mean [SD]) | 65.2 (11.7) | 64.9 (11.3) | 65.8 (12.7) | <0.001 |
| Female n (%) | 3,780 (27.4) | 2,666 (26.5) | 1,114 (29.9) | <0.001 |
| Past medical history n (%) |  |  |  |  |
| Depression | 3,594 (26.1) | 2,512 (25.0) | 1,082 (29.0) | <0.001 |
| Hypertension | 5,719 (41.5) | 4,143 (41.2) | 1,576 (42.2) | 0.28 |
| Ischaemic heart disease | 3,669 (26.6) | 2,651 (26.4) | 1,018 (27.3) | 0.29 |
| Previous MI | 2,230 (16.2) | 1,621 (16.1) | 609 (16.3) | 0.78 |
| Previous Revascularisation | 1,290 (9.4) | 922 (9.2) | 368 (9.9) | 0.22 |
| Diabetes | 3,223 (23.4) | 2,529 (25.2) | 694 (18.6) | <0.001 |
| Chronic kidney disease | 167 (1.2) | 92 (0.9) | 75 (2.0) | <0.001 |
| Heart failure | 1,974 (14.3) | 1,428 (14.2) | 546 (14.6) | 0.53 |
| Ischaemic stroke | 911 (6.6) | 627 (6.2) | 284 (7.6) | 0.004 |
| PVD | 857 (6.2) | 566 (5.6) | 291 (7.8) | <0.001 |
| Atrial fibrillation | 1,476 (10.7) | 1,010 (10.0) | 466 (12.5) | <0.001 |
| ACS during index | 9,576 (69.5) | 7,266 (72.3) | 2,310 (61.9) | <0.001 |
| *LLT post PCI n (%)* |  |  |  | <0.001 |
| None | 896 (6.5) | 351 (3.5) | 545 (14.6) |  |
| High statin | 7,774 (56.4) | 5,815 (57.9) | 1,959 (52.5) |  |
| Non-high-intensity statin | 4,690 (34.0) | 3,562 (35.4) | 1,128 (30.2) |  |
| Statin & other | 285 (2.1) | 216 (2.1) | 69 (1.8) |  |
| Other LLT | 136 (1.0) | 106 (1.1) | 30 (0.8) |  |

ACS: acute coronary syndrome; LLT: lipid lowering therapy; MI: myocardial infarction; PCI: percutaneous coronary intervention; PVD: peripheral vascular disease; Other includes ezetimibe and/or fibrate
