# Supplementary material for: Achievement of European Society of Cardiology/European Atherosclerosis Society lipid targets in very high-risk patients: Influence of depression and sex
Source: PLoS One. 2022 Feb 25;17(2):e0264529. doi: 10.1371/journal.pone.0264529 (PMC8880762; doi:10.1371/journal.pone.0264529)
Supplement: S4 Table — (DOCX) [file pone.0264529.s008.docx]

**S4 Table. Directed acyclic graph guided binary logistic regression for estimating the effect of depression on odds of having a documented lipid level during follow-up.**

| **Covariate** | **Odds ratio** | **95% C.I.** | **p** |
| --- | --- | --- | --- |
| Diabetes | 1.50 | 1.37-1.65 | <0.001 |
| Deprivation index |  |  | 0.19 |
| 1 (most deprived) | 0.92 | 0.81-1.03 |  |
| 2 | 0.95 | 0.84-1.07 |  |
| 3 | 1.01 | 0.89-1.14 |  |
| 4 | 1.06 | 0.93-1.20 |  |
| 5 (least deprived) | REF |  |  |
| Depression | 0.81 | 0.74-0.88 | <0.001 |
