# Supplementary material for: Achievement of European Society of Cardiology/European Atherosclerosis Society lipid targets in very high-risk patients: Influence of depression and sex
Source: PLoS One. 2022 Feb 25;17(2):e0264529. doi: 10.1371/journal.pone.0264529 (PMC8880762; doi:10.1371/journal.pone.0264529)
Supplement: S5 Table — Directed acyclic graph guided binary logistic regression estimating the effect of depression on achieving ESC/EAS 2016 (A) low density lipoprotein, (B) non-high-density lipoprotein and (C) triglyceride targets during follow-up. (DOCX) [file pone.0264529.s009.docx]

**S5 Table. Directed acyclic graph guided binary logistic regression estimating the effect of depression on achieving ESC/EAS 2016 (A) low density lipoprotein, (B) non-high-density lipoprotein and (C) triglyceride targets during follow-up.**

A:

| **Covariate** | **Odds ratio** | **95% C.I.** | **p** |
| --- | --- | --- | --- |
| Age | 1.01 | 1.00-1.01 | 0.007 |
| Female | 0.57 | 0.52-0.63 | <0.001 |
| Diabetes | 1.28 | 1.16-1.41 | <0.001 |
| Hypertension | 0.92 | 0.84-1.00 | 0.053 |
| Deprivation index |  |  | 0.31 |
| 1 (most deprived) | 1.05 | 0.92-1.20 |  |
| 2 | 1.00 | 0.87-1.14 |  |
| 3 | 0.97 | 0.85-1.11 |  |
| 4 | 1.11 | 0.97-1.28 |  |
| 5 (least deprived) | REF |  |  |
| Depression | 0.83 | 0.75-0.92 | <0.001 |

B:

| **Covariate** | **Odds ratio** | **95% C.I** | **p** |
| --- | --- | --- | --- |
| Age | 1.02 | 1.01-1.02 | <0.001 |
| Female | 0.64 | 0.56-0.74 | <0.001 |
| Diabetes | 0.91 | 0.79-1.04 | 0.17 |
| Hypertension | 0.77 | 0.68-0.87 | <0.001 |
| Deprivation index |  |  | 0.31 |
| 1 (most deprived) | 1.09 | 0.91-1.31 |  |
| 2 | 0.91 | 0.76-1.10 |  |
| 3 | 0.95 | 0.79-1.14 |  |
| 4 | 1.04 | 0.86-1.27 |  |
| 5 (least deprived) | REF |  |  |
| Depression | 0.75 | 0.65-0.86 | <0.001 |

C:

| **Covariate** | **Odds ratio** | **95% C.I.** | **p** |
| --- | --- | --- | --- |
| Age | 1.03 | 1.02-1.04 | <0.001 |
| Female | 1.00 | 0.88-1.15 | 0.95 |
| Diabetes | 0.44 | 0.39-0.50 | <0.001 |
| Ischaemic stroke | 0.69 | 0.55-0.86 | 0.001 |
| Deprivation index |  |  | 0.12 |
| 1 (most deprived) | 0.78 | 0.64-0.94 |  |
| 2 | 0.86 | 0.71-1.05 |  |
| 3 | 0.85 | 0.70-1.04 |  |
| 4 | 0.90 | 0.73-1.11 |  |
| 5 (least deprived) | REF |  |  |
| Depression | 0.67 | 0.59-0.76 | <0.001 |
