# Supplementary material for: Achievement of European Society of Cardiology/European Atherosclerosis Society lipid targets in very high-risk patients: Influence of depression and sex
Source: PLoS One. 2022 Feb 25;17(2):e0264529. doi: 10.1371/journal.pone.0264529 (PMC8880762; doi:10.1371/journal.pone.0264529)
Supplement: S6 Table — Full mutually adjusted binary logistic regression identifying variables associated with achieving ESC/EAS 2019 targets during follow-up for (A) LDL-C, (B) non-HDL-C and (C) triglycerides. (DOCX) [file pone.0264529.s010.docx]

**S6 Table. Full mutually adjusted binary logistic regression identifying variables associated with achieving ESC/EAS 2019 targets during follow-up for (A) LDL-C, (B) non-HDL-C and (C) triglycerides**.

A:

| **Covariate** | **Odds ratio** | **95% C.I.** | **p** |
| --- | --- | --- | --- |
| Age | 1.02 | 1.01-1.02 | <0.001 |
| Female | 0.56 | 0.49-0.63 | <0.001 |
| Diabetes | 1.64 | 1.45-1.86 | <0.001 |
| Contemporary acute coronary syndrome | 1.58 | 1.38-1.81 | <0.001 |
| Hypertension | 1.00 | 0.89-1.11 | 0.98 |
| Chronic kidney disease | 1.56 | 0.91-2.68 | 0.10 |
| Heart failure | 0.87 | 0.75-1.02 | 0.09 |
| Ischaemic stroke | 0.89 | 0.71-1.12 | 0.31 |
| Peripheral vascular disease | 0.69 | 0.54-0.90 | 0.005 |
| Atrial fibrillation | 0.95 | 0.79-1.14 | 0.56 |
| Lipid lowering therapy (LLT) |  |  | <0.001 |
| No LLT | REF |  |  |
| High statin | 7.40 | 4.85-11.31 |  |
| Low statin | 3.08 | 2.01-4.72 |  |
| Other | 0.63 | 0.21-1.88 |  |
| Statin + other | 3.24 | 1.75-5.98 |  |
| Deprivation index |  |  | 0.48 |
| 1 (most deprived) | 1.08 | 0.92-1.27 |  |
| 2 | 0.96 | 0.81-1.13 |  |
| 3 | 1.04 | 0.88-1.22 |  |
| 4 | 1.10 | 0.93-1.30 |  |
| 5 (least deprived) | REF |  |  |
| Depression | 0.92 | 0.81-1.04 | 0.18 |

B:

| **Covariate** | **Odds ratio** | **95% C.I.** | **p** |
| --- | --- | --- | --- |
| Age | 1.03 | 1.03-1.04 | <0.001 |
| Female | 0.58 | 0.50-0.68 | <0.001 |
| Diabetes | 1.13 | 0.97-1.32 | 0.11 |
| Contemporary acute coronary syndrome | 1.67 | 1.42-1.98 | <0.001 |
| Hypertension | 0.87 | 0.76-1.00 | 0.046 |
| Chronic kidney disease | 1.13 | 0.52-2.44 | 0.76 |
| Heart failure | 0.80 | 0.65-0.98 | 0.034 |
| Ischaemic stroke | 0.96 | 0.73-1.26 | 0.78 |
| Peripheral vascular disease | 0.62 | 0.45-0.85 | 0.003 |
| Atrial fibrillation | 1.09 | 0.87-1.36 | 0.47 |
| Lipid lowering therapy (LLT) |  |  | <0.001 |
| No LLT | REF |  |  |
| High statin | 10.47 | 6.36-17.22 |  |
| Low statin | 3.99 | 2.40-6.63 |  |
| Other | 0.43 | 0.10-1.94 |  |
| Statin + other | 2.30 | 1.05-5.04 |  |
| Deprivation index |  |  | 0.54 |
| 1 (most deprived) | 1.01 | 0.82-1.23 |  |
| 2 | 0.90 | 0.74-1.10 |  |
| 3 | 0.87 | 0.71-1.06 |  |
| 4 | 0.93 | 0.76-1.14 |  |
| 5 (least deprived) | REF |  |  |
| Depression | 0.79 | 0.68-0.92 | 0.002 |

C:

| Covariate | Odds ratio | 95% C.I. | p |
| --- | --- | --- | --- |
| Age | 1.02 | 1.02-1.03 | <0.001 |
| Female | 0.87 | 0.79-0.96 | 0.005 |
| Diabetes | 0.53 | 0.48-0.59 | <0.001 |
| Contemporary acute coronary syndrome | 1.30 | 1.18-1.43 | <0.001 |
| Hypertension | 0.81 | 0.74-0.88 | <0.001 |
| Chronic kidney disease | 0.64 | 0.41-1.01 | 0.05 |
| Heart failure | 0.92 | 0.81-1.04 | 0.18 |
| Ischaemic stroke | 0.82 | 0.69-0.99 | 0.034 |
| Peripheral vascular disease | 0.72 | 0.60-0.87 | 0.001 |
| Atrial fibrillation | 1.17 | 1.01-1.36 | 0.041 |
| Lipid lowering therapy (LLT) |  |  | <0.001 |
| No LLT | REF |  |  |
| Fibrate or N3 | 0.88 | 0.47-1.61 |  |
| Statin | 1.93 | 1.60-2.34 |  |
| Statin + other | 1.56 | 1.11-2.18 |  |
| Deprivation index |  |  | <0.001 |
| 1 (most deprived) | 0.71 | 0.62-0.81 |  |
| 2 | 0.77 | 0.67-0.88 |  |
| 3 | 0.82 | 0.71-0.93 |  |
| 4 | 0.85 | 0.73-0.98 |  |
| 5 (least deprived) | REF |  |  |
| Depression | 0.72 | 0.65-0.80 | <0.001 |
