# Supplementary material for: Achievement of European Society of Cardiology/European Atherosclerosis Society lipid targets in very high-risk patients: Influence of depression and sex
Source: PLoS One. 2022 Feb 25;17(2):e0264529. doi: 10.1371/journal.pone.0264529 (PMC8880762; doi:10.1371/journal.pone.0264529)
Supplement: S7 Table — Directed acyclic graph guided binary logistic regression for estimating the effect of depression on achieving ESC/EAS 2019 (A) LDL-C, (B) non-HDL-C and (C) triglyceride targets during follow-up. (DOCX) [file pone.0264529.s011.docx]

**S7 Table. Directed acyclic graph guided binary logistic regression for estimating the effect of depression on achieving ESC/EAS 2019 (A) LDL-C, (B) non-HDL-C and (C) triglyceride targets during follow-up.**

A:

|  | **Odds ratio** | **95% C.I.** | **p** |
| --- | --- | --- | --- |
| Age | 1.01 | 1.00-1.01 | 0.002 |
| Female | 0.56 | 0.50-0.64 | <0.001 |
| Diabetes | 1.37 | 1.22-1.54 | <0.001 |
| Hypertension | 0.93 | 0.84-1.04 | 0.20 |
| Deprivation index |  |  | 0.46 |
| 1 (most deprived) | 1.08 | 0.92-1.27 |  |
| 2 | 1.00 | 0.85-1.17 |  |
| 3 | 1.09 | 0.93-1.28 |  |
| 4 | 1.13 | 0.96-1.33 |  |
| 5 (least deprived) | REF |  |  |
| Depression | 0.88 | 0.78-1.00 | 0.046 |

B:

|  | **Odds ratio** | **95% C.I.** | **p** |
| --- | --- | --- | --- |
| Age | 1.02 | 1.02-1.03 | <0.001 |
| Female | 0.59 | 0.51-0.68 | <0.001 |
| Diabetes | 0.91 | 0.79-1.05 | 0.20 |
| Hypertension | 0.80 | 0.70-0.91 | 0.001 |
| Deprivation index |  |  | 0.85 |
| 1 (most deprived) | 1.05 | 0.87-1.26 |  |
| 2 | 0.97 | 0.80-1.18 |  |
| 3 | 0.96 | 0.80-1.16 |  |
| 4 | 1.05 | 0.86-1.28 |  |
| 5 (least deprived) | REF |  |  |
| Depression | 0.74 | 0.64-0.86 | <0.001 |

C:

|  | **Odds ratio** | **95% C.I.** | **p** |
| --- | --- | --- | --- |
| Age | 1.02 | 1.01-1.02 | <0.001 |
| Female | 0.86 | 0.78-0.95 | 0.002 |
| Diabetes | 0.48 | 0.44-0.53 | <0.001 |
| Ischaemic stroke | 0.78 | 0.65-0.93 | 0.005 |
| Deprivation index |  |  | <0.001 |
| 1 (most deprived) | 0.71 | 0.63-0.82 |  |
| 2 | 0.78 | 0.68-0.90 |  |
| 3 | 0.84 | 0.73-0.96 |  |
| 4 | 0.87 | 0.75-1.00 |  |
| 5 (least deprived) | REF |  |  |
| Depression | 0.70 | 0.64-0.78 | <0.001 |
